# Tree pruner: An efficient tool for selecting data from a biased genetic database

**DOI:** 10.1186/1471-2105-12-51

**Published:** 2011-02-09

**Authors:** Mohan Krishnamoorthy, Pragneshkumar Patel, Mira Dimitrijevic, Jonathan Dietrich, Margaret Green, Catherine Macken

**Affiliations:** 1 Theoretical Division, Los Alamos National Laboratory, Los Alamos, NM, USA; 2 Northrop Grumman Health IT, Rockville, MD, USA; 3 Genentech Inc, 1 DNA Way, South San Francisco, California, USA

## Abstract

**Background:**

Large databases of genetic data are often biased in their representation. Thus, selection of genetic data with desired properties, such as evolutionary representation or shared genotypes, is problematic. Selection on the basis of epidemiological variables may not achieve the desired properties. Available automated approaches to the selection of influenza genetic data make a tradeoff between speed and simplicity on the one hand and control over quality and contents of the dataset on the other hand. A poorly chosen dataset may be detrimental to subsequent analyses.

**Results:**

We developed a tool, *Tree Pruner*, for obtaining a dataset with desired evolutionary properties from a large, biased genetic database. Tree Pruner provides the user with an interactive phylogenetic tree as a means of editing the initial dataset from which the tree was inferred. The tree visualization changes dynamically, using colors and shading, reflecting Tree Pruner actions. At the end of a Tree Pruner session, the editing actions are implemented in the dataset.

Currently, Tree Pruner is implemented on the Influenza Research Database (IRD). The data management capabilities of the IRD allow the user to store a pruned dataset for additional pruning or for subsequent analysis. Tree Pruner can be easily adapted for use with other organisms.

**Conclusions:**

Tree Pruner is an efficient, manual tool for selecting a high-quality dataset with desired evolutionary properties from a biased database of genetic sequences. It offers an important alternative to automated approaches to the same goal, by providing the user with a dynamic, visual guide to the ongoing selection process and ultimate control over the contents (and therefore quality) of the dataset.

## Background

Infectious pathogens such as influenza virus and HIV have large databases of genetic data. In spite of efforts to sequence broadly representative viruses, bias still emerges from the substantial contribution of data from specialized sources, such as infection outbreaks, vaccine candidate selection or clinical studies. When databases are large and imbalanced, they pose a substantial challenge to selection of a dataset with desired evolutionary properties, such as evolutionary representation or shared genotype. An obvious selection approach is to control for relevant epidemiological variables. However, the resulting dataset may not have the desired properties, particularly when organisms are readily transmitted. For example, two identical (or almost identical) human influenza A viruses can be collected from hosts separated by thousands of miles. An alternative selection approach is to use an alignment of candidate nucleotide or protein sequences for visualizing genetic relationships. However, alignments are viewed as deviations from a reference sequence, making it easy to see when a sequence is similar to the reference sequence, but difficult to visualize the degree of similarity among non-reference sequences.

When data sets are imbalanced, clustering into groups of similar sequences is a powerful adjunct to the selection process. An example of such a capability for influenza sequences is SmartBLAST [1]. SmartBLAST uses a query sequence to search the database for other sequences within a close distance, as measured by BLAST. SmartBLAST then collapses the ordered BLAST hits into clusters of similar sequences and chooses a representative of each cluster to comprise a dataset for subsequent analysis. Currently, SmartBLAST operates on protein sequences and the degree of similarity that determines cluster membership is preset. Performance is achieved by pre-computing pairwise BLAST similarity scores between all pairs of sequences in the influenza database.

SmartBLAST is well suited for selecting a coarse representation from a very large data set. For example, SmartBLAST with a recent (2009) influenza A hemagglutinin (H1) query sequence efficiently selects 120 representatives from 3000+ hemagglutinin (H1) sequences from viruses collected between 1918 and the present. However, there are tradeoffs for the simplicity and efficiency of the automation of SmartBLAST. For example, SmartBLAST does not allow the user to control the degree of similarity required for clustering. In the above example of SmartBLAST, all 2009 pandemic H1 sequences fell into a single cluster. Further, SmartBLAST operates on protein sequences. Therefore, similarity in SmartBLAST does not guarantee closeness in a nucleotide-based phylogenetic tree. If a cluster can be equally well represented by multiple sequences, the user cannot choose from alternatives based on relevant epidemiological or other variables. Automation also denies the user the possibility of recognizing and culling data with incorrect details. SmartBLAST does not allow the user to define the domain within which selections are to be made. For example, a user may be interested in only sequences from a specific host species. Such epidemiological control is not possible in SmartBLAST. For the above reasons, we believe that SmartBLAST generates data sets well suited to exploratory data analysis, but possibly not well suited for detailed analysis of specific evolutionary events.

To select a dataset with the desired evolutionary properties, selection from a phylogeny of the candidate data provides a direct, and therefore efficient, approach. The *adaptive tree visualization *capability [2] of the Influenza Virus Resource [3] can vary the amount of detail presented in a phylogenetic tree display. Tree-distance between sequences is used to automatically collapse sub-trees to a degree of detail controlled by the user. However, the visualization does not provide a mechanism for producing a dataset to match the tree as ultimately visualized. As with SmartBLAST, the automation of the adaptive tree visualization has tradeoffs for the user, who gives up control over the selection of the representatives of a sub-tree.

We developed a tool, *Tree Pruner*, for obtaining a dataset with desired evolutionary properties from a large, biased genetic database. Tree Pruner produces a high-quality dataset by giving the user complete control over the data selection process. Thus, Tree Pruner trades off some of the simplicity and speed of automation in the interest of greater quality of the final data selection. Data selection is carried out by interaction with a display of the phylogeny of a large selection of data from a domain of interest. Custom Tree Pruner commands allow the user to fade-out or accentuate tips or sub-trees in the tree display to represent de-selection or selection of data to be included in the final dataset. Thus, at all times the user has a view of the approximate tree of selected sequences only. Because Tree Pruner gives the user complete control over the choice of evolutionarily representative sequences, it fosters selection of high-quality datasets. When Tree Pruner is connected to data management capabilities, such as those of the Influenza Research Database, the user can save an edited dataset for further editing or subsequent analysis.

## Implementation

### Requirements

The development of Tree Pruner was subject to both scientific and technical requirements. The critical scientific requirements were (a) an interactive phylogenetic tree display that allows the user to read taxon labels regardless of the size of the tree, so that the user can curate the data; and (b) data management support for successive rounds of editing. The existing "working set" capabilities of the Influenza Research Database (IRD: [4,5]) were used to satisfy (b). A number of existing code bases for interactive tree displays (including Archaeopteryx [6], TreeDyn, [7], Phy.Fi [8] and PhyloExplorer [9]) were examined for their potential to satisfy (a). Since Tree Pruner would be web-enabled, Java code was preferred in order to achieve platform-independence. Additionally, because IRD is a free, open web site, only source code available free or through open-source agreements, or whose licensing requirements, if any, would permit redistribution of modified source code was considered. Out of the available options, Archaeopteryx [6] was selected. Source code for Archaeopteryx is available on SourceForge (http://sourceforge.net/projects/forester-atv/).

### Overview of a Tree Pruner session

Users begin a Tree Pruner session with queries (possibly constrained by epidemiological variables) of the IRD and save selected results in a stored working set. When a Tree Pruner session is initiated on this working set, the IRD server infers a basic tree from the working set. Tree Pruner does not need a high-quality (and computationally expensive) tree; it is effective for a simple and quickly computed tree, leaving any refined phylogenetic inference to the final, pruned data set. When pruning is complete, Tree Pruner sends to IRD a list of the unique identifiers of those records to be deleted from the working set. Since Tree Pruner is intended to work on large datasets, we chose to carry out pruning on the client side, to minimize the burden on performance due to communications with the server.

### Tree Pruner applet

The Tree Pruner code modifies 10 classes of Archaeopteryx and adds 20 new classes. Modifications to Archaeopteryx provide: (a) custom functions in the control panel, used for editing; (b) painting of the display to represent editing actions; and (c) custom interactions with the applet for AutoSave, termination, crash recovery and opening a sub tree in a new window. Tree Pruner also supports export of PNG or JPEG images to the user's desktop.

Input to Tree Pruner is a modified Newick tree file. The standard Newick format is modified to use two labels for each taxon. One label is a strain name, which, for influenza virus, contains essential epidemiological information, including geographical location, year and host species from which the sample was collected. This information is important for curation during the editing process. However, since a given influenza virus can be sequenced multiple times, the second taxon label is an identifier unique to a particular sequence (generally a GenBank accession number). This unique label is essential for ensuring that a tip in the phylogeny and a record in the working set are correctly matched when editing the dataset. Tree Pruner parses the input Newick tree file so that the accession numbers, which are generally uninformative, are omitted from the tree display. If the user re-roots the tree, using the appropriate Archaeopteryx command, Tree Pruner models the new hierarchical relationships among taxa.

### Applet-server communications

Communications between the Tree Pruner applet and the server comply with the REST architecture (http://www.ics.uci.edu/~fielding/pubs/dissertation/rest_arch_style.htm). HTTP GET and POST commands pass information between the server and the client.

After generating the modified Newick file, IRD stores the file in a web-accessible location on its server. The user is then presented with a web page that contains the Tree Pruner applet. One of the parameters passed to the applet is the URL of the Newick file on the IRD server. Tree Pruner uses HTTP GET to retrieve the Newick file. Another parameter is the URL for making service requests using HTTP POST. Tree Pruner uses HTTP POST to send data to the IRD server in JavaScript Object Notation (JSON) format to call services. The data sent contains an action describing the type of service needed and data that acts as input to that service. The following services can be requested: commit - apply changes made in Tree Pruner to the working set and store in the IRD database; save/auto-save - store the current state of Tree Pruner (represented in JSON), but do not delete anything from the working set; discard - remove any stored Tree Pruner state, but do not delete anything from the working set; lock - mark a working set as locked to prevent conflicts that could arise if multiple sessions attempt to edit the same working set; unlock - reverse the locking of a working set.

If a session becomes suspended, Tree Pruner supports auto-recovery by using HTTP GET to request the state that was most recently stored. Tree Pruner then makes its user interface consistent with the stored state.

The server locks the working set during a Pruner session; the set is unlocked once the user commits or discards all changes. If the user exits from Tree Pruner without committing or discarding all saved changes, the working set will remain locked until a new Tree Pruner session is initiated and saved changes are committed or discarded. Locking a working set ensures that the 1:1 relationship between taxa in the tree and records in the working set is not corrupted.

Tree Pruner was designed flexibly to enable interaction with server software other than IRD. To support Tree Pruner, server software must implement an API to provide the services listed above; there are no further technology or language restrictions on the server software.

## Results

Tree Pruner is an efficient, visual editing capability for obtaining a dataset of genetic sequences with desired properties, such as evolutionary representation or shared genotype. Importantly, it provides the user curatorial control over the final selection of sequence data. While it is currently used with the large, biased influenza sequence database, it can be implemented for other viral genetic databases, such as those for HIV and HCV.

### Overview of Tree Pruner

The two editing functions of Tree Pruner, *Keep *and *Remove/Restore*, act in complementary modes to edit a phylogenetic tree (and consequently edit a dataset). *Keep *is particularly suited to selection of a small subset of a dataset. *Remove/Restore *is particularly suited to fine-tuning a dataset by removal of just a few sequences. During a Tree Pruner session, editing actions are represented on the tree by changes in the color of branches and labels. At the end of a Tree Pruner session, the editing actions are committed to the original data set, resulting in the removal of sequences corresponding to deselected tips in the tree.

Editing actions are selected by custom additions to the Archaeopteryx drop-down menu for actions on nodes. Custom buttons on the Archaeopteryx control panel, such as Discard All or Commit Changes, translate editing actions into changes to the dataset.

### Example of editing using Tree Pruner

**Figure 1:**
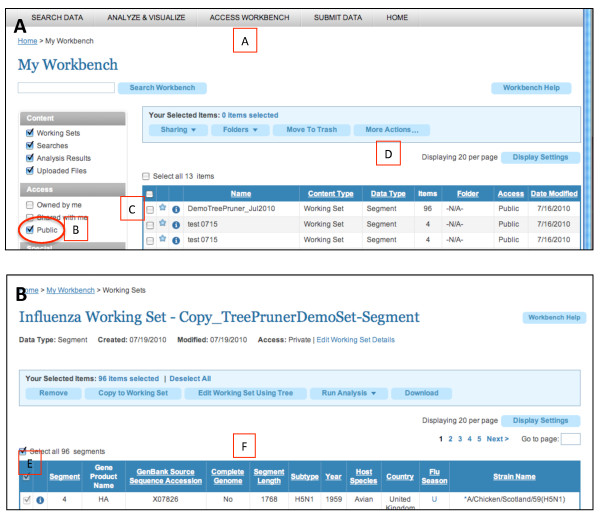
**Accessing Tree Pruner**. A working set for testing Tree Pruner is available on the Influenza Research Database (http://www.fludb.org). To test Tree Pruner, starting in panel 1A: (A) create a login to access data on the workbench; (B) list public working sets; (C) select the DemoTreePruner_Jul2010 set using the check box; (D) make a private copy, using the copy function from "More Actions". (Public working sets cannot be edited.) Display the contents of this copy by clicking the "i" button to the left of its name. Then, continuing in panel 1B: (E) select all records in the working set, and (F) click "Edit Working Set Using Tree" button.

The following illustrations of Tree Pruner are based on an initial dataset of all instances of sequences of the hemagglutinin (HA) gene from influenza A (H5N1) viruses that were collected in the period 1900 - 2000. A minimum length of 900 (out of a maximum of 1790) nucleotides was required for inclusion in this initial dataset. A search of the Influenza Research Database (IRD) [4,5], conducted on March 7 2010, yielded 96 sequences, which were stored in a working set called DemoTreePruner on the IRD server. Instructions for accessing this dataset to test Tree Pruner are given in Figure 1. (Alternatively, a static version of Tree Pruner with a pre-loaded tree of 953 NP sequences from seasonal influenza A (H1N1) viruses, together with an equine NP sequence as an outgroup, is available at the Influenza Sequence Database, http://www.flu.lanl.gov.)

Tree Pruner is launched from the working set, and automatically infers a phylogeny of the sequences in the set. (The IRD infers a maximum likelihood tree under the HKY model of evolution, using PhyML [10,11].) Tree Pruner then opens an Archaeopteryx applet, labeled with the name of the working set (DemoTreePruner) and the name of the gene (Segment 4), and displays this tree.

The user can carry out multiple *Keep *and/or *Remove *editing sessions. Before switching from one edit action to the other, the user must save or discard all edits of the current type. This ensures that the keep/remove status of all "untouched" taxa is defined. The user can end a Tree Pruner session by committing changes. Then taxa marked for removal in the tree will be removed from the dataset, thus maintaining a 1:1 relationship between the dataset and tree display.

#### (i) *Keep *function

**Figure 2:**
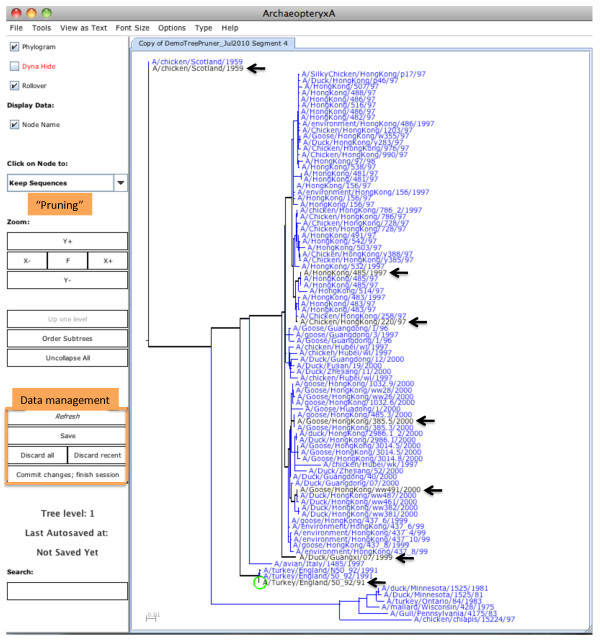
**Editing a dataset using *Keep***. The phylogenetic tree contains all sequences in the DemoTreePruner dataset (obtained using instructions in Figure 1). Black labels and lines indicate seven taxa that have been tagged for retention in the dataset (black labels are indicated by black arrows). The remaining sequences are currently active, indicated by the blue labels and lines: these can be selected by future *Keep *actions. At the end of a *Keep *session, the user can *Save *these actions and switch to a *Remove/Restore *session; any unselected (blue) tips will be colored grey. If no further editing actions are performed, sequences corresponding to black labels in the tree will comprise the edited dataset. Other features: the green circle indicates the most recently touched node; custom Tree Pruner data management functions are indicated; the custom *Keep *action is selected in the pull-down menu indicated; the tab at the top of the tree gives the name of the working set that is being edited.

When a user clicks on a node at the tip of the tree, or at the root of a sub-tree, the label(s) of the selected tip (sub-tree) is (are) written in black, designating inclusion in the final dataset. All non-selected labels are written in blue. Blue tips are "active," meaning they can be selected by future actions, but are not currently designated for inclusion in the final dataset. Branches are also colored; black branches lead to sequences selected for inclusion in the dataset; blue branches lead to sequences whose status is undetermined. (See Figure 2.)

#### (ii) *Remove/Restore *function

**Figure 3:**
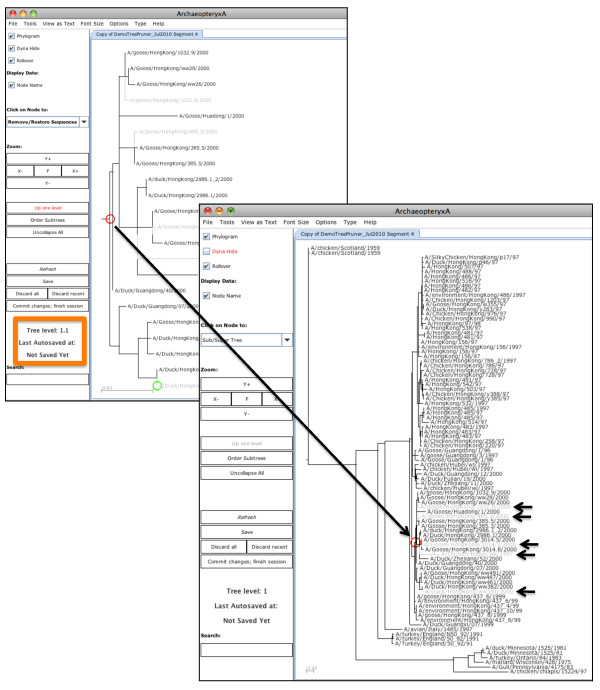
**Editing a dataset using *Remove/Restore *and sub-trees**. The right tree contains all sequences in the DemoTreePruner dataset (obtained using instructions in Figure 1); the left tree is the sub-tree based at the node marked by a red circle in the right tree. The label in the orange box gives the sub-tree level. Grey taxa labels and lines indicate five sequences that have been tagged for removal by editing the sub-tree. Actions in the sub-tree have been mapped back to the parent tree by using the *Refresh *button. At the end of a *Remove/Restore *session, the user can *Save *these actions and switch to *Keep *actions. If no further editing actions are performed, sequences corresponding to black labels in the tree will comprise the edited dataset.

When a user clicks on a node at the tip of the tree, or at the root of a sub-tree, the color of the label(s) of the selected tip (sub-tree) switches between black and grey. All non-selected labels remain black. Black tips are "active" and can be removed by future actions. Alternatively, clicking on a grey node is a *Restore *action. *Restore *will change the tip label(s) and relevant branches from grey to black. (See Figure 3.)

#### (iii) Handling large trees

A key scientific requirement of Tree Pruner was to enable the user to view taxon labels, even in large trees. Archaeopteryx offers three features for viewing large trees. "Dynamic hiding" displays a subset only of taxon labels in order to squeeze a large tree into the window. Zooming allows reading of all taxon labels, but loses the overall tree structure. Viewing a sub-tree allows reading of all taxon labels in a sub-tree, but loses the context of editing because the sub-tree replaces the full tree in the window. Archaeopteryx was modified so that Tree Pruner displays a sub-tree in a separate window, side-by-side with the display of the complete tree. Multiple levels of sub-tree are permitted. Pruning actions are mapped among windows by the *Refresh *action. Thus, a sub-tree can be pruned while viewing its context.

#### (iv) Concluding a Tree Pruner session

The user may use *Commit *to exit from Tree Pruner; all taxa tagged for removal (i.e., drawn in grey in the phylogeny) will be removed from the working set. The tree used to edit the dataset is removed from the server. To perform further editing, Tree Pruner infers a tree from the revised dataset, thereby retaining a 1:1 correspondence between the dataset and the tree display.

#### (v) Crash recovery

*AutoSave *is run every 10 minutes after the most recent user-instigated *Save*. If the server or client crashes during a pruning session, Tree Pruner can resume from the most recent (*Auto*)*Save*.

## Discussion

Selecting a dataset of sequences with specific evolutionary properties from a large, biased genetic database is problematic. Selection by inspection and editing of a phylogeny of candidate sequences is an obvious approach. Tools such as TreeDyn [7] and Archaeopteryx [6] allow manual editing of a phylogenetic display; the Influenza Virus Resource's [3] adaptive tree visualization [2] uses an algorithm to automatically edit a phylogenetic display. However, none of these tools can translate editing actions into data selection.

Tree Pruner was designed for selecting data from the influenza genetic sequence database. Tree Pruner uses a manual editing process to avoid the potential of automatic editing to select poor quality data. The intuitive editing actions of Tree Pruner allow the user to quickly converge on a final data set with the required evolutionary properties. Tree Pruner uses sub-trees to facilitate reading the labels on a large tree, thus fostering curation of the final data. Communications between the Tree Pruner applet and the server translate editing of the phylogeny into selection of data from the database.

The size of the dataset that can be edited by Tree Pruner is limited by the capacity of the server to infer a phylogeny for the initial superset of data and not by the capacity of Archaeopteryx, which can display trees of 5,000 - 10,000 taxa without significant degradation of performance. Currently, PhyML is used to infer trees for Tree Pruner; PhyML works reliably for trees with 1,000 - 2,000 taxa. Thus, Tree Pruner is relevant for typical large influenza virus sequence analyses.

Tree Pruner can be easily adapted for data selection from large genetic databases of other organisms such as HIV, provided the appropriate software for applet-server communications is used, and the database has a means of storing a dataset.

## Conclusions

Tree Pruner is an efficient tool for selecting a curated dataset with the required evolutionary properties from a large, biased database of genetic sequences.

## Availability and Requirements

Project name: Tree Pruner

Code availability: http://github.com/caml/Tree-Pruner/

Link to full implementation: http://www.fludb.org/

Link to static demonstration: http://www.flu.lanl.gov

Link to user guide: http://www.fludb.org/brcDocs/tutorials/TreeEditor.pdf

http://www.flu.lanl.gov/

Operating systems Platform-independent if browser is Java-compatible

Programming language: Java

Other requirements: A code-signing certificate is required to allow exporting of graphics onto a user's desktop.

License: L-GPL

## Authors' contributions

MK, PP, MD wrote the Tree Pruner code; MG guided the technical development; JD and the Northrop Grumman team implemented the code on the Influenza Research Database and contributed to the manuscript. CM provided the scientific guidance and wrote the manuscript. All authors read and approved the final manuscript.

## References

[B1] Zaslavsky L Tatusova T Mining the NCBI influenza sequence database: adaptive grouping of BLAST results using precalculated neighbor indexing PLoS Curr Influenza 2009 RRN1124 10.1371/currents.RRN1124 PMC2771650 20029662

[B2] Zaslavsky L Bao Y Tatusova TA Visualization of large influenza virus sequence datasets using adaptively aggregated trees with sampling-based subscale representation BMC Bioinformatics 2008 9 237 10.1186/1471-2105-9-237 18485197 PMC2416652

[B3] The Influenza Virus Resource http://www.ncbi.nlm.nih.gov/genomes/FLU/FLU.html

[B4] The Influenza Research Database http://www.fludb.org/

[B5] Squires B Macken C Garcia-Sastre A Godbole S Noronha J Hunt V Chang R Larsen CN Klem E Biersack K Scheuermann RH BioHealthBase: informatics support in the elucidation of influenza virus host pathogen interactions and virulence Nucleic Acids Res 2008 36 Database D497 503 17965094 10.1093/nar/gkm905 PMC2238987

[B6] Archaeopteryx http://www.phylosoft.org/archaeopteryx/

[B7] Chevenet F Brun C Banuls AL Jacq B Christen R TreeDyn: towards dynamic graphics and annotations for analyses of trees BMC Bioinformatics 2006 7 439 10.1186/1471-2105-7-439 17032440 PMC1615880

[B8] Fredslund J PHY.FI: fast and easy online creation and manipulation of phylogeny color figures BMC Bioinformatics 2006 7 315 10.1186/1471-2105-7-315 16792795 PMC1513607

[B9] Ranwez V Clairon N Delsuc F Pourali S Auberval N Diser S Berry V PhyloExplorer: a web server to validate, explore and query phylogenetic trees BMC Evol Biol 2009 9 108 10.1186/1471-2148-9-108 19450253 PMC2695458

[B10] Guindon S Delsuc F Dufayard JF Gascuel O Estimating maximum likelihood phylogenies with PhyML Methods Mol Biol 2009 537 113 137 full_text 19378142 10.1007/978-1-59745-251-9_6

[B11] Guindon S Gascuel O A simple, fast, and accurate algorithm to estimate large phylogenies by maximum likelihood Syst Biol 2003 52 5 696 704 10.1080/10635150390235520 14530136

